# Thirty-three long-term survivors after cytoreductive surgery in patients with peritoneal metastases from colorectal cancer: a retrospective descriptive study

**DOI:** 10.1186/s12957-021-02145-1

**Published:** 2021-01-28

**Authors:** Yasuyuki Kamada, Koya Hida, Haruaki Ishibashi, Shouzou Sako, Akiyoshi Mizumoto, Masumi Ichinose, Naveen Padmanabhan, Shinya Yoshida, Yutaka Yonemura

**Affiliations:** 1 Department of Surgery, Graduate School of Medicine, Kyoto University, 54, Shogoin-Kawahara-Cho, Sakyo-Ku, Kyoto, Japan; 2 NPO to support Peritoneal Surface Malignancy Treatment, Japanese/Asian School of Peritoneal Surface Oncology, Kyoto, Japan; 3 Department of Regional Cancer Therapy, Peritoneal Surface Malignancy Center, Kishiwada Tokushukai Hospital, Kishiwada, Japan; 4 Department of Regional Cancer Therapy, Peritoneal Surface Malignancy Center, Kusatsu General Hospital, Shiga, Japan; 5 Department of Surgical Oncology, Apollo Cancer Institute, Chennai, India

**Keywords:** Peritoneal metastasis, Colorectal cancer, Long-term survivors, Cytoreductive surgery, HIPEC

## Abstract

**Background:**

Cytoreductive surgery (CRS) combined with hyperthermic intraperitoneal chemotherapy (HIPEC) improves survival in selected patients with peritoneal metastasis (PM) from colorectal cancer (CRC). However, little has been reported on characteristics and clinical course of long-term survivors with CRC-PM beyond 5 years. The objective of this study was to identify the clinical and oncological features affecting long-term survival of CRC-PM after comprehensive treatment.

**Methods:**

Between January 1990 and April 2015, CRC-PM patients who underwent CRS with or without HIPEC in two Japanese tertiary hospitals were analyzed. Clinicopathological parameters and therapeutic details for long-term survivors (patients surviving ≥ 5 years after CRS) were described and compared with those for non-survivors (patients surviving < 5 years).

**Results:**

The study identified 236 patients with CRC-PM who underwent CRS, with a median follow-up period of 2.5 years. Thirty-three patients (14.0%) were considered as long-term survivors. Compared with non-survivors, long-term survivors had a lower median peritoneal cancer index (PCI) [4 (1–27) vs 9 (0–39), *p* < 0.001]. Complete cytoreduction (CCR-0) was achieved in all long-term survivors, with a significantly higher rate [33/33 (100%) vs 141/203 (69.8%), *p* < 0.001]. Metachronous onsets of PM were more frequently observed in the long-term survivor group [26/33 (78.8%) vs 103/203 (50.3%), *p* = 0.018]. Regarding histopathology, long-term survivors more frequently had mucinous adenocarcinoma than non-survivors [8/33 (24.2%) vs 27/203 (13.3%)] and less likely exhibited poorly differentiated or signet ring cell carcinoma [2/33 (6.1%) vs 48/203 (23.7%)] (*p* < 0.001).

**Conclusions:**

One in seven patients with CRC-PM achieved the long-term milestone after CRS. A long-term survival was associated with the presence of low PCI, CCR-0, metachronous onset, and mucinous histology.

## Introduction

Colorectal cancer (CRC) represents the third most frequent cancer diagnosis and second most frequent cause of cancer-related mortality throughout the world [1]. An estimated 2–4% of patients have synchronous peritoneal metastasis (PM) [2–4], and approximately 20% develop metachronous PM during the course of their disease [5]. PM was traditionally considered a terminal event, and patients were palliated with chemotherapy or minor surgical procedures. The overall estimated survival in untreated cases was 6 months [2, 6, 7], whereas with contemporary systemic chemotherapy, the median overall survival (OS) has been prolonged to 20 months [8–10].

Over the past two decades, several centers worldwide have adopted extensive cytoreductive surgery (CRS) and hyperthermic intraperitoneal chemoperfusion (HIPEC), aiming for cure in patients with PM. With this approach, long-term survival has been reported in CRC-PM, with a median OS of 20–63 months [11–20]. A randomized trial proved the survival benefit in CRC-PM patients undergoing CRS/HIPEC over systemic chemotherapies [21], although the study encountered the criticism that the study cohort included appendiceal primary carcinoma, which is biologically different from CRC.

Selecting the appropriate candidates for CRS and HIPEC is vital to achieve long-term survival in CRC patients with PM. Several studies revealed the various features associated with survival benefits in detail [22–26], although there has been little research which focused on long-term survivors diagnosed with CRC-PM. Additionally, 5-year survival probabilities were estimated in most of the past studies.

The data on oncologic outcomes in CRC-PM patients surviving beyond 5 years are sparse. Therefore, the aim of this study is to describe the characteristics of long-term survivors among patients with PM from CRC, with the goal of finding clinical and pathological factors associated with survival longer than 5 years after CRS.

## Methods

### Patients

This was a retrospective study of CRC-PM patients who had undergone CRS with or without HIPEC in two Japanese tertiary hospitals. Inclusion criteria were the following: (1) histopathologically proven PM from CRC and (2) treated with CRS between January 1990 and April 2015. Exclusion criteria were the following: (1) PM from appendiceal carcinoma, (2) treated with only systemic chemotherapy, and (3) followed-up less than 5 years but alive at the last encounter. We identified and stratified into the two groups based on OS: long-term survivors (who lived at least 5 years after the first CRS) and non-survivors (who died within 5 years). We then described the characteristics of long-term survivors in detail, and moreover, compared the factors among the two groups.

Patients who had a recurrence-free survival (RFS) ≥ 5 years after the last operation for metastases were considered as cured patients. Because there is no official definition of a long-term survival and cure in CRC-PM patients, we used an OS and RFS of ≥ 5 years as our criteria.

### Surgical treatment and intraperitoneal chemotherapy

The suitability of CRS and HIPEC was decided based on the consensus of a multidisciplinary team. Patients as follows were not eligible for CRS and HIPEC: ECOG performance status of more than 2, serious comorbidities, unresectable metastasis other than PM, and severe PM not amenable to curative intent resection.

All patients were treated by cytoreduction according to the Sugarbaker technique after intraperitoneal exploration [27]. The extent of intraoperative tumor volume was measured using the peritoneal cancer index (PCI) described by Jaquet and Sugarbaker [28]. PCI after January 1997 was prospectively recorded, while in the cases before 1997, estimated PCI was measured using operation records and pathological reports. The intent of CRS was to remove all visible intraperitoneal disease (completeness of cytoreduction). At the completion of surgery, the completeness of cytoreduction (CCR) score was recorded [28]: CCR-0 (no residual macroscopic tumor), CCR-1 (residual tumor deposits < 2.5 mm in diameter), and CCR-2 (residual tumor deposits > 2.5 mm in diameter). After completing CRS (CCR-0 or CCR-1), HIPEC was performed with the open coliseum technique with 4 L of physiological saline (0.9%) as perfusate [29]. The target temperature was 42.5–43.5 °C, and treatment time was 30–60 min. The drug regimen varied based on patient factors and prior neoadjuvant therapies. Commonly, 5-fluorouracil (5-FU), oxaliplatin, mitomycin C, and cisplatin were used alone or in combination. HIPEC was not performed in patients induced to poor general conditions after CRS due to a severe surgical stress (e.g., massive bleeding, prolonged operative time) and/or patient comorbidities. Major surgical complications within 30 days after CRS were defined as any intra- or extra-abdominal event with a grade ≥ III according to the Clavien-Dindo classification [30].

### Data collection

Between January 1997 and April 2015, the prospective institutional database was searched to identify eligible patients. A standard data form before 1997 was retrospectively completed. The data comprised the following: onset of PM, primary tumor location, histology, lymph node metastasis, KRAS, BRAF, PCI, CCR, HIPEC drug, postoperative complications, preoperative and postoperative chemotherapy, site of first recurrence, RFS, reoperation for recurrence, PCI and CCR at reoperation, and OS. The primary tumor located in the cecum, ascending colon, or transverse colon was defined as right-sided colon cancer, and those located in the splenic flexure, descending colon, or sigmoid colon were defined as left-sided colon cancer. Follow-up involved a clinical examination, tumor marker measurement, and imaging when required, every 3 months in the first 2 years, every 6 months for the next 3 years, and annually thereafter until any oncological event. The details of recurrence and consequent management details were noted, and follow-up frequency was modified based on the last event.

This retrospective study was approved by the ethics review committee for clinical studies of our institution. Our study was performed in accordance with the ethical guidelines of the Declaration of Helsinki. The patients involved in this study provided written informed consent authorizing the use and disclosure of their protected health information.

### Statistical analysis

The closing date of follow-up for this study was the 30 April 2020. Using the Kaplan−Meier method, OS was calculated from the date of the first CRS for PM until the patient’s death or last follow-up, and RFS was measured from the date of CRS until the date of first recurrence or last follow-up, including death. Continuous variables were given as median (range). Categorical data are given as frequencies and proportions. Chi-squared test or Fisher’s exact test was used in categorical variables where appropriate, and Wilcoxon two sample test in continuous variables. A two tailed *p* value < 0.05 was considered significant. Variables proved to be significant by univariate analysis were included in multivariate logistic regression model. All statistical analyses were conducted by JMP statistical software version 14 (SAS Institute, Cary, NC).

## Results

### Study population

Between January 1990 and April 2015, 236 patients underwent CRS with or without HIPEC for CRC-PM, with a median follow-up time of 2.5 years. The median survival time was 1.6 years. The cohort predominantly consisted of females (55.1%), and the median age was 59 years (range, 24–80). The median PCI of this cohort was 9 (range, 0–39). Of these patients, 33 patients (14.0%) were classified as long-term survivors. Laparoscopic exploration or exploratory laparotomy was not performed in most cases.

### Description of long-term survivors

#### Patients’ characteristics

The demographics of long-term survivors are summarized in Table 1. The group consisted of 21 women and 12 men, with a median age of 59 (range, 33–75) years. The onset of PM was synchronous in 7 patients and metachronous in 26 patients. The primary tumor was located in the right colon in 17 patients and in the left colon in 16 patients. None of the patients had a primary tumor in the rectum. Histological diagnoses were well to moderately differentiated tubular adenocarcinoma, mucinous adenocarcinoma (MC), and signet ring cell carcinoma (SRCC) in 24, 7, and 2 cases, respectively. Lymph node metastases were observed on pathology in 21 patients. The status of mutation in KRAS and BRAF were examined in 15 and 5 patients, respectively. KRAS mutations were observed in 4/15 (26.7%) and BRAF in 0/5 (0%). The median PCI in long-term survivors was 4 (range, 1–27). Categorizing PCI in this group, 28 patients (84.8%) had PCI < 10, 4 (12.1%) had PCI 10–19, and 1 patient (3.1%) had PCI ≥ 20. The median PCI of the cured subgroup was 2 (range, 1–8). Among the 16 cured patients, 15 patients (93.8%) had PCI 1–5.

**Table 1:** Patient and treatment demographics of long-term survivors

Case no.	Age	Gender	Onset of	Primary tumor	Histology	Lymph node	KRAS	BRAF	PCI	CCR	HIPEC	Postoperative	Preoperative	Postoperative
			PM	Location		Metastasis	Mutation	Mutation	at CRS	at CRS		Complication (G ≥ III)	CT	CT
1	67	F	Metachronous	Left	sig	Negative	WT	WT	4	0	5-FU + OX	None	Modern CT, IP	Not done
2	66	F	Metachronous	Right	tub	Positive	NE	NE	5	0	CDDP+MMC	Intraabdominal	Modern CT	Modern CT
3	50	F	Metachronous	Right	tub	Negative	WT	WT	4	0	5-FU + OX	None	Modern CT	5-FU
4	65	F	Metachronous	Left	muc	Positive	Mutated	NE	16	0	Not done	None	IP	5-FU
5	71	F	Metachronous	Right	sig	Positive	WT	NE	5	0	CDDP+MMC	None	Modern CT	Modern CT
6	50	M	Metachronous	Left	tub	Positive	WT	WT	7	0	CDDP+MMC	None	5-FU	Modern CT
7	66	F	Metachronous	Left	tub	Positive	Mutated	NE	1	0	CDDP+MMC	None	5-FU	5-FU
8	48	F	Metachronous	Left	tub	Positive	WT	NE	6	0	CDDP+MMC	Extraabdominal	5-FU	Modern CT
9	57	F	Metachronous	Right	tub	Positive	NE	NE	4	0	CDDP+MMC	None	Modern CT	5-FU
10	74	M	Synchronous	Right	muc	Negative	NE	NE	12	0	Not done	None	5-FU	5-FU
11	59	M	Metachronous	Left	tub	Positive	WT	NE	10	0	5-FU + OX	None	Modern CT	5-FU
12	63	M	Metachronous	Left	tub	Negative	WT	NE	2	0	Not done	None	Modern CT	Modern CT
13	61	F	Metachronous	Right	tub	Positive	NE	NE	3	0	CDDP+MMC	None	Modern CT	Modern CT
14	47	M	Metachronous	Right	tub	Negative	WT	NE	3	0	CDDP+MMC	None	Modern CT	5-FU
15	59	F	Metachronous	Left	tub	Positive	NE	NE	1	0	5-FU + OX	None	Modern CT	Modern CT
16	69	F	Metachronous	Left	muc	Negative	NE	NE	12	0	CDDP+MMC	Intraabdominal	IP	5-FU
17	36	F	Synchronous	Right	muc	Negative	WT	WT	27	0	CDDP+MMC	Extraabdominal	Modern CT	5-FU
18	48	M	Synchronous	Left	tub	Positive	NE	NE	2	0	CDDP+MMC	None	Not done	Not done
19	35	M	Synchronous	Left	tub	Positive	NE	NE	2	0	5-FU + MMC	None	Modern CT	Modern CT
20	72	F	Metachronous	Right	tub	Positive	NE	NE	2	0	5-FU + OX	None	Modern CT	5-FU
21	66	F	Metachronous	Left	tub	Negative	NE	NE	2	0	Not done	None	Modern CT	5-FU
22	57	F	Synchronous	Right	tub	Positive	WT	WT	2	0	CDDP+MMC	None	Modern CT	Modern CT
23	75	F	Metachronous	Left	tub	Positive	Mutated	NE	3	0	5-FU + OX	None	5-FU	5-FU
24	67	M	Metachronous	Left	tub	Negative	NE	NE	4	0	Not done	Intraabdominal	Modern CT	Not done
25	49	M	Metachronous	Right	tub	Positive	NE	NE	4	0	5-FU + OX	None	Modern CT	Not done
26	66	M	Metachronous	Left	tub	Positive	NE	NE	4	0	Not done	None	Not done	Not done
27	58	F	Metachronous	Left	tub	Positive	NE	NE	8	0	Not done	None	Modern CT	Modern CT
28	62	F	Metachronous	Right	tub	Positive	Mutated	NE	4	0	CDDP+MMC	None	Modern CT	5-FU
29	37	F	Metachronous	Right	muc	Negative	WT	NE	2	0	CDDP+MMC	None	Modern CT	5-FU
30	33	M	Metachronous	Right	muc	Negative	NE	NE	1	0	CDDP+MMC	None	Modern CT	Modern CT
31	59	F	Synchronous	Right	muc	Positive	NE	NE	3	0	CDDP+MMC	None	IP	5-FU
32	33	M	Metachronous	Right	tub	Negative	NE	NE	1	0	CDDP+MMC	None	5-FU, IP	5-FU
33	57	F	Synchronous	Right	tub	Positive	NE	NE	2	0	CDDP+MMC	None	Modern CT	Modern CT

Abbreviations: *CCR* completeness of cytoreduction, *CDDP* cisplatin, *CRS* cytoreductive surgery, *CT* chemotherapy, *F* female, *HIPEC* hyperthermic intraperitoneal chemotherapy, *IP* intraperitoneal chemotherapy, Left** left-sided colon, *M* male, *MMC* mitomycin C, *muc* mucinous adenocarcinoma, *NE* not examined, *no.* number, *OX* oxaliplatin, *PCI* peritoneal cancer index, Right* right-sided colon, *sig* signet ring cell carcinoma, *tub* well to moderately differentiated tubular adenocarcinoma, *WT* wild type

*Right represents the right-sided colon cancer defined as any tumor in the cecum, ascending colon, and transverse colon

**Left represents the left-sided colon cancer defined as any tumor in the descending colon and sigmoid colon

#### Treatment factors

Table 1 shows the treatment factors of long-term survivors. Most of the patients received systemic chemotherapy: 28 (84.8%) of the 33 patients received preoperative and postoperative chemotherapy. Modern chemotherapy agents (fluorinated pyrimidine plus oxaliplatin or irinotecan, ± bevacizumab or panitumumab) were used in 22 patients (66.7%) receiving preoperative regimens and in 12 patients (36.4%) receiving postoperative regimens. Five patients underwent preoperative intraperitoneal chemotherapy with cisplatin and/or docetaxel.

CCR-0 was achieved in all 33 patients, and 26 patients received HIPEC. Seven patients did not undergo HIPEC because of deterioration of their general condition secondary to massive bleeding during the CRS procedure. The HIPEC regimens were cisplatin plus mitomycin C in 18 patients, 5-FU plus oxaliplatin in 7 patients, and mitomycin C plus 5-FU in 1 patient. Among the long-term survivors, there was no significant difference in OS and RFS between HIPEC and non-HIPEC groups (OS, *p* = 0.302; RFS, *p* = 0.445).

Three patients of 33 long-term survivors experienced major intra-abdominal complications, and two experienced major extra-abdominal complications (grades III or IV).

#### Patient outcomes

**Fig. 1:**
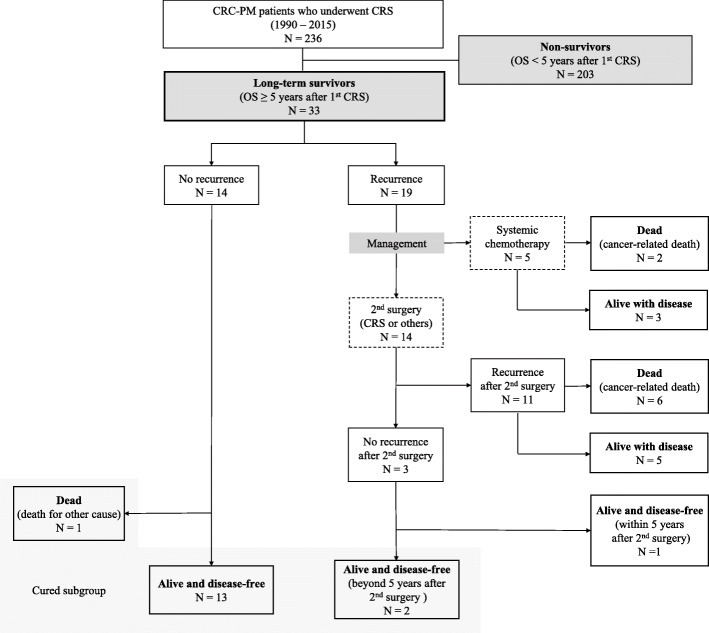
Flow diagram of patient enrollment. Abbreviations: CRC colorectal cancer, CRS cytoreductive surgery, OS overall survival, PM peritoneal metastasis

Patients’ prognoses are presented in Table 2, and a flow chart is shown in Fig. 1. The 14 patients who did not develop recurrence after the first CRS and the 2 patients who survived at least 5 years after the last operation without a second recurrence were considered “cured.” The patient characteristics between cured and non-cured patients are compared in Table 3. Among the long-term survivors, 5 patients survived beyond 10 years after the first CRS.

**Table 2:** Postoperative course of long-term survivors

Case no.	Recurrence after CRS	RFS (year)	The sites of first recurrence	Reoperation for recurrence	PCI at reoperation	CCR at reoperation	Second recurrence	OS (year)	Status (dead or alive)	Prognosis (cured or non-cured)
1	Yes	0.8	Abdominal wall	Metastasectomy	0	0	Yes	5.1	Alive	Non-cured
2	Yes	2.9	Peritoneum	CRS/HIPEC	7	0	Yes	5.2	Dead	Non-cured
3	Yes	3.2	Abdominal wall	Not done	—	—	—	5.3	Alive	Non-cured
4	Yes	2.0	Abdominal wall	Metastasectomy	0	0	Yes	5.5	Alive	Non-cured
5	Yes	2.1	Peritoneum	Not done	—	—	—	5.9	Alive	Non-cured
6	Yes	2.2	Abdominal wall	Metastasectomy	2	0	Yes	6.0	Alive	Non-cured
7	Yes	0.7	Liver	Metastasectomy	0	0	Yes	6.0	Dead	Non-cured
8	Yes	4.2	Peritoneum	CRS/HIPEC	2	1	Yes	6.2	Dead	Non-cured
9	Yes	2.7	Lung	Metastasectomy	0	0	No	6.3	Alive	Non-cured
10	Yes	4.6	Lymph node	Not done	—	—	—	6.8	Dead	Non-cured
11	Yes	2.6	Peritoneum, lymph node	CRS/HIPEC	5	0	Yes	7.6	Alive	Non-cured
12	Yes	1.5	Lymph node	Not done	—	—	—	7.7	Dead	Non-cured
13	Yes	2.2	Abdominal wall	Metastasectomy	3	0	Yes	7.9	Dead	Non-cured
14	Yes	5.6	Peritoneum, kidney	CRS	3	0	Yes	8.5	Dead	Non-cured
15	Yes	7.4	Peritoneum	Not done	—	—	—	9.1	Alive	Non-cured
16	Yes	2.0	Peritoneum	CRS	14	2	Yes	9.8	Dead	Non-cured
17	Yes	2.7	Peritoneum, abdominal wall	CRS/HIPEC	4	0	Yes	10.7	Alive	Non-cured
18	No	5.3	—	—	—	—	—	5.3	Alive	Cured
19	No	5.3	—	—	—	—	—	5.3	Alive	Cured
20	No	5.5	—	—	—	—	—	5.5	Alive	Cured
21	No	6.0	—	—	—	—	—	6.0	Dead^b^	Cured
22	No	6.8	—	—	—	—	—	6.8	Alive	Cured
23	No	6.9	—	—	—	—	—	6.9	Alive	Cured
24	No	6.9	—	—	—	—	—	6.9	Alive	Cured
25	No	7.1	—	—	—	—	—	7.1	Alive	Cured
26	No	7.8	—	—	—	—	—	7.8	Alive	Cured
27	No	8.8	—	—	—	—	—	8.8	Alive	Cured
28	No	9.6	—	—	—	—	—	9.6	Alive	Cured
29	No	9.9	—	—	—	—	—	9.9	Alive	Cured
30	Yes	4.6	Peritoneum	CRS/HIPEC	8	0	No^a^	10.2	Alive	Cured
31	No	12.1	—	—	—	—	—	12.1	Alive	Cured
32	No	12.3	—	—	—	—	—	12.3	Alive	Cured
33	Yes	1.6	Liver, lung	Metastasectomy	0	0	No^a^	28.8	Alive	Cured

Abbreviations: *CCR* completeness of cytoreduction, *CRS* cytoreductive surgery, *G* Clavien-Dindo classification grade, *no.* number, *OS* overall survival, *PCI* peritoneal cancer index, *RFS* recurrence-free survival

^a^The re-recurrence-free intervals were at least 5 years after the 2nd surgery

^b^Non-cancer-related death

**Table 3:** Clinicopathological characteristics between patients cured or non-cured

	Total (*n* = 33)	Non-cured (*n* = 17)	Cured (*n* = 16)
**Age, year, median (range)**	59 (33–75)	59 (36–74)	58.5 (33–75)
**Gender,** ***n***			
Male	12	5	7
Female	21	12	9
**Timing of peritoneal metastasis,** ***n***			
Synchronous	7	2	5
Metachronous	26	15	11
**Primary tumor location,** ***n***			
Right colon	17	8	9
Left colon	16	9	7
Rectum	0	0	0
**Histology,** ***n***			
tub	24	11	13
muc	7	4	3
sig	2	2	0
**Lymph node metastasis,** ***n***			
Negative	12	7	5
Positive	21	10	11
**PCI at CRS, median (range)**	4 (1–27)	5 (1–27)	2 (1–8)
0–5	25	10	15
6–10	4	3	1
11–15	2	2	0
16–20	1	1	0
≥ 21	1	1	0
**Completeness of cytoreduction**			
CCR-0	33	17	16
CCR-1 to 3	0	0	0
**HIPEC,** ***n***			
Yes	26	14	12
No	7	3	4
**Postoperative complication (*G*** **≥ 3)**			
Yes	5	4	1
No	28	13	15
**Preoperative systemic chemotherapy,** ***n***			
Yes	28	15	13
No	5	2	3
**Preoperative intraperitoneal chemotherapy,** ***n***			
Yes	5	3	2
No	28	14	14
**Postoperative chemotherapy,** ***n***			
Yes	28	16	12
No	5	1	4
**Recurrence,** ***n***			
Yes	19	17	2
No	14	0	14

Abbreviations: *CCR* completeness of cytoreduction, *CRS* cytoreductive surgery, *G* Clavien-Dindo classification grade, *HIPEC* hyperthermic intraperitoneal chemotherapy, *muc* mucinous adenocarcinoma, *PCI* peritoneal cancer index, *sig* signet ring cell carcinoma, *tub* well-differentiated tubular adenocarcinoma

Tumor recurrence occurred in 19/33 cases at a median of 2.6 (range, 0.7–7.4) years. Among 19 patients with recurrence, 16 received HIPEC at the first CRS. The site of first recurrence included the peritoneum (*n* = 9), abdominal wall (*n* = 6), lymph nodes (*n* = 3), liver (*n* = 2), lung (*n* = 2), and kidney (*n* = 1). In this group of 19 patients with recurrence after CRS, 5 were treated with palliative systemic therapy and 14 with a second surgical procedure; 7 in metastasectomy, 5 in CRS/HIPEC, and 2 in CRS. Median PCI in the second operation was 2 (range, 0–14). Twelve patients achieved CCR-0, and one each achieved CCR-1 and CCR-2. In the group who underwent secondary cytoreduction or metastasectomy, 11 developed a second recurrence. Among these 11 patients with re-recurrences, 6 patients died of a cancer-related cause, and 5 patients were alive with disease at the last follow-up.

### Comparison between long-term survivors and non-survivors

The patient characteristics of 33 long-term survivors and 203 non-survivors are compared in Table 4. Metachronous onsets of PM were more frequent in the long-term survivor group [26/33 (78.8%) vs 103/203 (50.3%), *p* = 0.018]. Long-term survivors more frequently had MC than non-survivors [8/33 (24.2%) vs 27/203 (13.3%)], and in addition, less likely presented with poorly differentiated or SRCC [2/33 (6.1%) vs 48/203 (23.7%)] (*p* < 0.001). The median PCI was significantly lower in long-term survivors [4 (1–27) vs 9 (0–39), *p* < 0.001]. CCR-0 was achieved in 174 of total 236 CRC-PM patients, with a significantly higher rate in long-term survivors [33/33 (100%) vs 141/203 (69.8%), *p* < 0.001]. Lymph node metastases were observed in 20/33 long-term survivors (60.6%) and in 136/203 non-survivors (67.0%) (*p* = 0.553). HIPEC administration did not differ significantly between long-term survivors and non-survivors [26/33 (78.8%) vs 142 (70.0%), *p* = 0.407].

**Table 4:** Clinicopathological characteristics of long-term survivors and non-survivors

	Long-term survivors (*n* = 33)	Non-survivors (*n* = 203)	Univariate	Multivariate	
			*p* value	OR (95% CI)	*p* value
**Age, year, median (range)**	59 (33–75)	58 (24–80)	0.429		
**Gender,** ***n*** **(%)**			0.287		
Male	12 (36.4)	94 (46.3)			
Female	21 (63.6)	109 (53.7)			
**Timing of PM,** ***n*** **(%)**			**0.003**		**0.018**
Synchronous	7 (21.2)	100 (49.3)		Reference	
Metachronous	26 (78.8)	103 (50.7)		3.10 (1.14, 8.39)	
**Primary tumor location,** ***n*** **(%)**			0.753		
Right colon	16 (48.5)	102 (50.3)			
Left colon	17 (51.5)	98 (48.3)			
Rectum	0 (0)	3 (1.5)			
**Histology,** ***n*** **(%)**			**0.036**		**< 0.001**
tub	23 (69.7)	128 (63.1)		11.86 (2.88, 48.82)	
muc	8 (24.2)	27 (13.3)		Reference	
por or sig	2 (6.1)	48 (23.7)		0.031 (0.004, 0.228)	
**Lymph node metastasis,** ***n*** **(%)**			0.553		
Negative	13 (39.4)	67 (33.0)			
Positive	20 (60.6)	136 (67.0)			
**PCI at CRS, median (range)**	4 (1–27)	9 (0–39)	**< 0.001**	0.001 (1.68E−05, 0.055)	**< 0.001**
**Completeness of cytoreduction,** ***n*** **(%)**			**< 0.001**		**< 0.001**
CCR-0	33 (100)	141 (69.8)		Reference	
CCR-1 to 3	0 (0)	61 (30.2)		8.19E + 07 (0.00, -)	
**HIPEC,** ***n*** **(%)**			0.407		
Yes	26 (78.8)	142 (70.0)			
No	7 (21.2)	61 (30.1)			
**Postoperative complocation (*G*** **≥ 3),** ***n*** **(%)**			0.199		
Yes	5 (15.2)	53 (26.2)			
No	28 (84.9)	150 (73.9)			
**Preoperative systemic chemotherapy,** ***n*** **(%)**			0.147		
Yes	28 (84.9)	190 (93.6)			
No	5 (15.2)	13 (6.4)			
**Postoperative chemotherapy,** ***n*** **(%)**			0.820		
Yes	27 (81.8)	158 (77.8)			
No	6 (18.2)	45 (22.2)			

Taken together, age, gender, primary tumor location, lymph node metastasis, HIPEC administration, postoperative complication (grade ≥ III), preoperative systemic chemotherapy, and postoperative chemotherapy were similar between the two groups. Univariate analysis demonstrated four variables (onset of PM, histology, PCI, CCR) to be significantly associated with OS ≥ 5 years. Multivariate analysis was carried out on these four factors, and all the factors were found to be significant factors for long-term survival.

## Discussion

In our cohort of patients with CRC-PM who underwent extensive CRS and perioperative chemotherapy including systemic and intraperitoneal chemotherapy, 14.0% (33/236) survived beyond 5 years. Sixteen of 33 patients remained recurrence-free more than 5 years after the last surgery for metastases and were considered “cured.” Additionally, 5 patients in this cohort survived more than 10 years. Our study proves that long-term survival and cured status are possible in an appropriately selected sub-set of patients with PM from CRC.

Despite the adoption of CRS and HIPEC in many centers worldwide, this approach is still met with criticism. One of the arguments against CRS and HIPEC is high morbidity and mortality risk of these procedures [31–33]. However, whether patients with PM from CRC can attain equivalent long-term survival with systemic therapy alone is doubtful [8–10]. A comprehensive approach with a combination of neoadjuvant systemic chemotherapy, CRS/HIPEC, and adjuvant systemic therapy may provide long-term survival in CRC-PM patients. This retrospective study was conducted to evaluate the characteristics of long-term survivors diagnosed with CRC-PM who underwent CRS with or without HIPEC. Kaplan-Meier survival analyses tend to overestimate survival rate due to the effect of censor before the end of observation. By contrast, results from this present study provide specific and actual information on long-term outcome for CRC-PM patients. Although we only analyzed selected patients, understanding the results of our study may help improve outcomes in CRC-PM patients.

First, OS in CRC-PM patients treated with CRS is strongly associated with achieving complete cytoreduction. Several studies showed that patients with complete cytoreduction (CCR-0 or CCR-1) have a better survival outcome than patients with incomplete cytoreduction (CCR-2 or CCR-3) [34–36]. Others reported survival differences between CCR-0 and CCR-1 in CRC-PM patients [37]. In our study, all 33 patients received CCR-0 resection, and CCR-0 was demonstrated to be an independent prognostic factor for long-term survival. These results reaffirm that complete cytoreduction with no macroscopic disease is important to achieve long-term survival.

Second, PCI, which describes the extent and distribution of peritoneal disease, is one of the most important prognostic indices. Several investigators have suggested that better outcomes are obtained after CRS and HIPEC with a PCI < 10 [38, 39], and worse survival with a PCI > 17 [40, 41]. In our study, the median PCI in long-term survivors was significantly lower than in non-survivors. Moreover, all patients in the cured subgroup had a PCI ≤ 8. However, it is also noteworthy that we identified a subset of long-term survivors with a PCI > 15. While these findings confirmed the notion that PCI can be useful to predict outcome, a high PCI cannot entirely exclude the possibility of long-term survival.

Third, the current study showed that histopathology of CRC was related to long-term survival. In the group of long-term survivors, 7 patients had MC, and 2 had SRCC. Histological differences between mucinous and non-mucinous regarding prognosis are controversial. Some investigators suggested that mucinous carcinoma patients had a worse prognosis [42, 43] while others did not [44, 45]. Interestingly, we identified MC as being associated with the actual long-term survival. Meanwhile, the negative impact of SRCC in CRC-PM has been described in multiple studies, with the median OS in these patients ranging from 7 to 13 months even if patients are treated with CRS and HIPEC [36, 46–49]. In this present study, a long-term survivor group less likely showed SRCC. However, two patients with SRCC achieved a 5-year survival. The proportion of SRCC patients who are eligible for CRS and consequently experience long-term survival is currently low. More detailed reporting and further research are required to identify potential long-term survivors.

Fourth, it was surprising to discover that the present study identified metachronous PM as being associated with long-term survival in patients with CRC-PM. A possible explanation is that disease progression of synchronous PM is considered aggressive. In contrast, two previous studies have shown that onset of PM does not affect OS [23, 50], but little evidence is available with regard to impact of timing when CRC-PM occurred. Information on onset of PM is preoperatively available while most prognostic factors for survival outcome in CRC-PM patients are determined in the operating room. Whether metachronous onset of colorectal PM or not might be helpful in predicting long-terms survival.

Fifth, patients who had lymph node metastases constituted more than one-half of long-term survivors and the subgroup of cured patients. It has been proposed that regional lymph node metastasis has a negative prognostic impact on survival [37, 51–53]. The recently developed COMPASS (colorectal peritoneal metastases prognostic surgical score) reported by Simkens et al. includes nodal status among the four clinical factors (PCI, nodal status, histology, and age) used to predict outcomes after CRS and HIPEC in CRC-PM [54]. However, lymph node meatastases in isolation cannot be considered an exclusion criterion [39]. With standardization of techniques for total mesorectal excision and complete mesocolic excision, which removes tumors en bloc with lymphatics, local recurrence has decreased [55, 56]. Among the long-term survivor group, the majority (21/33, 63.6%) of patients presented with lymph node metastases, and 10 of 21 patients were categorized into the cured subgroup. There was no statistically significant difference regarding nodal status between long-term survivors and non-survivors. At least, it is reasonable to support that CRC-PM patients with lymph node metastasis can achieve long-term survival and cure.

Finally, we found that HIPEC was not associated with long-term survival. The recent randomized control trial (PRODIGE-7) questioned the role of HIPEC with oxaliplatin in the clinical management of PM from CRC [57]. Although our findings are in agreement with the results of the trial, the present study does not necessarily support the ineffectiveness of HIPEC. We did not perform HIPEC in patients with poor general condition after CRS, due to a severe surgical stress and/or patient comorbidities. These patients without HIPEC tended to exhibit severe extent of peritoneal diseases and to have poor prognoses. Further studies should be required to elucidate whether HIPEC affects survival outcomes in patients with CRC-PM.

This study had several major limitations. First, because our study was retrospective in design, selection biases were introduced, due to the exclusion of patients with unresectable PM. Second, as a descriptive study, this current research lacked any comparison of control groups for statistical analysis of effectiveness of CRS/HIPEC. Third, the definition of long-term survival and cure is not officially defined and was based only on survival times. Finally, we may not have detected some other differences because the total number of long-term survivors is small. However, the data in this study allowed a detailed assessment of the clinical features among CRC-PM patients.

## Conclusions

Although CRC-PM is a challenging disease, one in seven patients actually achieved a 5-year survival after CRS. Low PCI, CCR-0, metachronous onset, and mucinous histology were associated with a higher likelihood of survivorship. It is important to continue identifying long-term survivors who enjoy the benefit of CRS.

## Data Availability

All data are available without restriction. Researchers can obtain data by contacting the corresponding author.

## Abbreviations

CCR: Complete cytoreduction
CRC: Colorectal cancer
CRS: Cytoreductive surgery
FU: Fluorouracil
HIPEC: Hyperthermic intraperitoneal chemotherapy
MC: Mucinous adenocarcinoma
OS: Overall survival
PCI: Peritoneal cancer index
PM: Peritoneal metastasis
RFS: Recurrence-free survival
SRCC: Signet ring cell carcinoma
